# Supplementary material for: Early bilingual immersion school program and cognitive development in French-speaking children: Effect of the second language learned (English vs. Dutch) and exposition duration (2 vs. 5 years)
Source: PLoS One. 2021 Oct 14;16(10):e0258458. doi: 10.1371/journal.pone.0258458 (PMC8516207; doi:10.1371/journal.pone.0258458)
Supplement: Attachment — Submitted filename: Response to Reviewer.docx [file pone.0258458.s002.docx]

**Response to Reviewers**

**We thank the reviewers and the editor for their constructive comments which allowed us to refine our manuscript.**

6. Review Comments to the Author

Please use the space provided to explain your answers to the questions above. You may also include additional comments for the author, including concerns about dual publication, research ethics, or publication ethics. (Please upload your review as an attachment if it exceeds 20,000 characters)

**Reviewer #1:**

Summary. This study tested for differences in three components of executive functioning (alerting, cognitive flexibility, and working memory) and arithmetic abilities in children (either 2nd or 5th grade) in three language groups: English immersed, Dutch immersed, and non- immersed French monolinguals. No differences between the three language groups were obtained in second grade, but in fifth grade the two immersed groups outperformed the monolingual controls on the cognitive flexibility task (but did not differ from each other). Only the Dutch immersed group outperformed the monolingual control in the working memory task. With respect to arithmetic, the Dutch immersed group outperformed the monolingual controls who, in turn, were better than the English immersed group.

In this revision the authors have been very responsive to the points raised by the reviewers and they deserve considerable credit for having done so. I would have no objection to the publication of this study as it matches the standards in the relevant literature.

However, I would say that more-of-the-same will not move the needle in resolving the many inconsistencies in this literature. The sample sizes are far too small, especially when participants cannot be randomly assigned to the conditions of interest. Furthermore, the children in the different language groups are taught by different teachers and have different peer cohorts. (Although the groups are matched on age, SES, and IQ and that’s great!)

Response: We fully agree that further studies should ideally include larger samples. We added this limitation and made reference to Brysbaert (2020), page 27 of the manuscript.

Some of the key results may be due to extraneous factors – consider the finding that only the Dutch immersed group outperformed the monolingual group in the working memory task. The authors spend a fair amount of time discussing why language similarity may sometimes lead to differences between the two immersion groups, but intuitively the differences in similarity are more subtle (see Paap, Darrow, Dalibar, & Johnson, 2015 for details) than the contrast between bilinguals and monolinguals. Yet the working memory results show no differences between French-English bilinguals and monolinguals.

Response: Concerning the advantage (or “boost”) observed for the working memory task in Dutch immersed children as compared to non-immersed and English immersed children, our main hypothesis is that the learning of languages characterized by different canonical syntactic structures – SVO and SOV – should may stimulate the ability to maintain different parts of a sequence of stimuli in a working memory task (see Amici et al., 2019): SVO structure languages would enhance the focus on final items and SOV structure language would enhance the focus on initial items. Hence, the combined learning of two languages with different structures could consequently lead to better overall recall performance in working memory tasks (better recall of first *and* last items of the sequence). This hypothesis needs however to be tested more directly. Another hypothesis is that this advantage could be related to the manipulation of two different number systems for the French-speaking children immersed in Dutch: a system with a unit-ten structure in Dutch and a system with a ten-unit structure in French. Particularly the Dutch structure will be challenging for French-speaking children as the linguistic order will be the opposite of the written numbers (e.g.: 21 = eenentwentig), and hence the number words need to be maintained in working memory the time they are fully processed and associated with the correct numbers while in French a more direct linear correspondence will operate (e.g., 21 = vingt-et-un)

The incoherent pattern of results gets worse when the present results are integrated with earlier results: “**It seems that the moment of appearance of the cognitive advantages, as well as the specific cognitive function(s) enhanced, vary in function of the language learned. Moreover, once the advantage appears, it may not be sustainable**. In English immersed children, advantage in selective auditory attention for example appears in first grade [3], not in second grade, reappears in third grade [2] and is not found later in CLIL schooling. The present study showed an advantage in cognitive flexibility only later in the schooling (fifth grade) confirming the outcomes of a previous study in Dutch CLIL context, that found no advantages in first, second, or third grades while advantages were found in sixth grade in cognitive flexibility and working memory[5].” This now you see it, now you don’t pattern is consistent with chronically underpowered studies. I simply have no confidence that an exact replication with sample sizes of 30 would produce the same pattern of interaction across tasks and language groups. Small sample sizes do not simply make it less likely to detect small real effects, they also make false positives more likely. I applaud Brysbaert’s 2020 recent plea (Bilingualism: Language & Cognition) for bilingual researchers to step up our game and recruit adequate sample sizes even when it is difficult and costly to do so. I suspect that closure will not come until we invest in a large N longitudinal study.

Response: Indeed, the effect size of the interaction is low as well as associated power. To try to qualify our point further, we added the statement 'if a cognitive advantage is demonstrated', line 482 to insist on the fact that it may not necessarily appear in other replication studies. However, we should note that since 2012, we conducted 7 studies in our lab with the same tasks (Kitap and Tab batteries) as used here and the same type of population (French speaking children immersed in Dutch or English Elementary schools), with sample sizes varying between 30 and 60 children per group. Of these studies, five found an advantage in one or more cognitive function(s) while two found no advantage. Thus, if the group differences observed in these studies would be statistical artefacts with the true effect being a no effect, then we would have expected a majority of studies showing no effect, and a few studies showing either an advantage or a disadvantage. However, the latter was never observed and the majority of studies showed an effect (although not always on the same measures). Hence, we think that a cognitive advantage could emerge over the course and context of CLIL schooling. Like mentioned above, we also added a reference of Brysbaert (2020), acknowledging that future studies should use larger sample sizes.

**Minor comments:**

p. 10 “The experimenters had to test the same number of immersed and non-immersed children.” How could this be if there are twice as many immersed participants as non-immersed?

Response: We corrected the sentence; it is the same number of children *in each language group*. Thank you.

p. 21. “anecdotic” should be anecdotal

Response: We changed the word, thank you!

p. 26. “Note however that some researchers suggested that there was a clear publication bias toward studies showing a bilingual advantage and suggested that this advantage do [does] not exist or is really small and/or task-dependent [51-53].” At the risk of tooting our own horn this would be a good additional study to cite here: Paap, Mason, Zimiga, Ayala-Silva, & Frost (2021). The alchemy of confirmation bias transmutes expectations into bilingual advantages: A tale of to new meta-analyses. QJEP, [73(8), 1290-1299](callto:73(8),%201290-1299).

Response: We added the study of Paap et al. (2021) as well as relevant references,

**Reviewer #2:**

I believe the manuscript is in a much better shape than in its original form, three very minor points:
p 16 line 347 - space missing('3 as' not '3as')

Response: This error has been corrected, thank you!

p 22 line 495/496 - I still somewhat stumble over the argument here - what would an alerting task requiring 'more profound cognitive processing' be?

Response: Future studies may use slightly more demanding tasks than the detection of simple visual stimuli such as an odd/even number judgement for example. The use of these more complex reaction time tasks would allow for a better characterization of the specificity the reaction time differences in tasks involving cognitive flexibility. Consequently, we added the example of an odd/even judgement task on page 23 that allows matching the groups on cognitive processing and not just on perceptive reaction time

p 26 line 580 - the shift in topic seems rather abrupt, I wonder whether a subheading would be good here

Response: We added the following subheading ‘arguments against a cognitive advantage of bilingualism’. Thank you for this suggestion.

**Other comments from the authors:**

1. All references have been checked for completeness and accuracy. Two references have been added and the reference De Wilde et al., 2017, was removed.
